# Microcirculation Improvement in Diabetic Foot Patients after Treatment with Sucrose Octasulfate-Impregnated Dressings

**DOI:** 10.3390/jcm12031040

**Published:** 2023-01-29

**Authors:** José Luis Lázaro-Martínez, Marta García-Madrid, Serge Bohbot, Mateo López-Moral, Francisco Javier Álvaro-Afonso, Yolanda García-Álvarez

**Affiliations:** 1 Diabetic Foot Unit, Clínica Universitaria de Podología, Facultad de Enfermería, Fisioterapia y Podología, Universidad Complutense de Madrid, Instituto de Investigación Sanitaria del Hospital Clínico San Carlos (IdISSC), 28040 Madrid, Spain; 2 Laboratoires URGO, 75116 Paris, France

**Keywords:** diabetic foot, neuroischaemic diabetic foot ulcer, microcirculation, sucrose octasulfate dressing, transcutaneous oxygen pressure

## Abstract

To assess the patients’ microcirculation evolution during the treatment with a sucrose octasulfate-impregnated dressing, fifty patients with neuroischaemic DFU treated with TLC-NOSF dressing were included in a prospective study between November 2020 and February 2022. TcpO_2_ values were measured on the dorsalis pedis or tibial posterior arteries’ angiosome according to the ulcer location. TcpO_2_ values were assessed at day 0 and every 4 weeks during 20 weeks of the follow-up or until the wound healed. A cut-off point of tcpO_2_ < 30 mmHg was defined for patients with impaired microcirculation. The TcpO_2_ values showed an increase between day 0 and the end of the study, 33.04 ± 12.27 mmHg and 40.89 ± 13.06 mmHg, respectively, *p* < 0.001. Patients with impaired microcirculation showed an increase in the tcpO_2_ values from day 0 to the end of the study (*p* = 0.023). Furthermore, we observed a significant increase in the TcpO_2_ values in the forefoot DFU (*p* = 0.002) and in the rearfoot DFU (*p* = 0.071), with no difference between the ulcer locations (*p* = 0.694). The local treatment with TLC-NOSF dressing improved the microcirculation in patients with neuroischaemic DFU, regardless of microcirculation status at the baseline, and in the forefoot, regardless of the location.

## 1. Introduction

Neuroischaemic diabetic foot ulcers (DFUs) have become the commonest ulcer type among patients with diabetes [1]. Foot tissues can become ischemic because of macrovascular disease (atherosclerosis); however, when we are evaluating the genesis of diabetic foot complications, not only macrovascular complications, but also the presence of microvascular complications seem to be important predictors for the development and the prognosis of the DFU [2]. The relationship between DFU and peripheral arterial disease (PAD) has been well recognized. Nevertheless, the role of impaired microcirculation is yet to be fully understood [3].

It has been demonstrated that microangiopathy may play a significant role in the pathogenesis of tissue breakdown, and it may become an important factor in the poor healing of wounds [3]. It is essential for skin nutrition, fluid homeostasis, thermoregulation, the provision of defense, and the repair of cells and cytokines following injury and infection. Consequently, impaired microcirculation will impact these essential processes [4]. Microangiopathy comprises detrimental changes in the nerve microvasculature’s structure and function, which in turn cause reduced endoneurial perfusion and hypoxia. Consequently, reducing the supply of oxygen to nerves and tissues causes a disturbance in the metabolism of cells, which significantly impedes the viability of tissues [3].

Several technological methods have enabled us to evaluate the microcirculation status, such as laser Doppler flowmetry, capillaroscopy, hyperspectral imaging, or transcutaneous oxygen pressure (tcpO_2_) techniques, among others [5]. Of them, the tcpO_2_ method is a non-invasive method that evaluates skin microcirculation and reflects tissue perfusion and oxygen delivery [6]. Additionally, tcpO_2_ is considered to better evaluate the microvascular function and its role in predicting foot ulcer healing and lower limb amputations [7].

Previous studies have documented the usefulness of different local therapies in microcirculation restoration and their potential effect on its improvement [8,9,10,11,12,13,14]. Of these, a recent pilot study has reported that sucrose octasulfate dressings (TLC-NOSF (Technology Lipido-Colloïd-Nano-OligoSaccharide Factor)) result in an increase in the skin oxygen pressure [15]. The power of this study remains poor due to the small sample size and the pilot study design. The development of these therapies that improve diabetic foot-impaired microcirculation could change the microvascular status of diabetic patients in the long-term follow-up period. Therefore, we aimed to assess the patients’ microcirculation improvement during a treatment with a sucrose octasulfate-impregnated dressing.

## 2. Materials and Methods

### 2.1. Subjects

A prospective study was conducted between November 2020 and February 2022 on 50 patients with DM who had non-infected neuroischaemic DFU in a specialized diabetic foot unit.

The inclusion criteria were: having confirmed type 1 or 2 DM, being aged >18 years old, presenting with non-infected neuroischemic DFU of grade IC or IIC, as defined by the University of Texas Diabetic Wound Classification System, having their glycemic control confirmed by an HbA1c (hemoglobin A1c) of ≤10% (85.8 mmol/mol) in the previous 3 months, and having a wound area surface size of between 1 to 30 cm^2^ at the moment of inclusion.

The exclusion criteria were: having a critical limb ischemia [16], end-stage renal disease or dialysis, the presence of edema from a vascular, renal, or cardiac disease, and patients with chronic obstructive pulmonary disease, which could alter oxygen saturation at a systemic level, patients who have suffered from a stroke in the last 3 months, those with acute Charcot foot, and those who had undergone surgical revascularization in the past 3 months before inclusion in the study.

This study was approved by the ethics committee of our teaching hospital in May 2020 (Code: 20/386-O_P). Before inclusion in this study, all of the patients provided their written informed consent according to the principles of the Declaration of Helsinki [17].

### 2.2. Clinical Assessment

The baseline patient assessment was carried out before the inclusion in the study, including diabetes type and duration, associated comorbidities, the HbA1c (%) values from the last blood test, and foot-related complications.

Neuropathy was diagnosed using a Biotensiometer and Semmes–Weinstein 5.07/10 g monofilament (Novalab Iberica, Madrid, Spain) [18]. Peripheral arterial disease (PAD) was diagnosed based on the distal pedal pulse palpation, ankle–brachial index (ABI), toe-brachial index (TBI), and tcpO_2_ [16].

The neuroischemic patients were defined as having an ankle–brachial index ABI of ≤0.9 and an ankle systolic blood pressure (ASBP) of ≥70 mmHg or a toe systolic blood pressure (TSBP) of at least 50 mmHg. In the patients with ABI > 0.9, we considered PAD when the toe-brachial pressure index (TBI) was <0.7 [15].

The patients were classified depending on the microcirculatory status at the baseline based on whether they had normal or impaired microcirculation. A cut-off point of tcpO_2_ < 30 mmHg was set for the patients with impaired microcirculation. For the patients with normal microcirculation, a cut-off point of >30 mmHg was set [19].

### 2.3. Wound Management and Follow-Up

All of the patients were dressed with a sucrose octasulfate dressing (UrgoStart Contact, 10 × 10 cm, Laboratories Urgo Medical, Paris, France). The patients came twice weekly to the outpatient clinic for dressing care until they were healed. Additionally, the patients received a high standard of care (SoC) with offloading, following the International Working Group of the Diabetic Foot (IWGDF) offloading Guidelines [20]. In addition, when it was necessary, sharp debridement was performed to remove the non-viable skin, including the peri-wound skin. Wound area surface, photographs, and Wollina scores were assessed during each study visit, monthly, and at the end of the study.

Microcirculation was measured using a tcpO_2_ TCM400 measuring device (Radiometer) following the angiosome concept according to the ulcer location [21]. The electrode was placed on the dorsalis pedis for the DFU located on the forefoot or in the posterior tibial artery for the DFU located on the midfoot or rearfoot. The values were recorded in mmHg after a calibrating them for a time of 10 min. The patients were supine during the examination, and they were asked not to move or speak. The TcpO_2_ values were assessed at the baseline (day 0) and every 4 weeks during 20 weeks of follow-up or until the wound healed.

The study’s main outcome was to evaluate the patients’ microcirculation improvement during the treatment with a sucrose octasulfate-impregnated dressing in patients with neuroischaemic diabetic foot ulcers.

### 2.4. Statistical Analysis

All of the statistical analyses were performed using the software package SPSS version 25.0 (IBM Corp. Released in 2017. IBM SPSS Statistics for Macintosh, Version 25.0. Armonk, NY, USA: IBM Corp.).

The assumption of normality of all of the continuous variables was verified using the Shapiro–Wilk test. The normally distributed variables (Shapiro–Wilk test with *p* ≥ 0.05) are reported as mean and standard deviations.

The categorical variables are reported as a frequency and percentage, while the continuous variables are reported as the mean ± standard deviation (SD; parametric distribution) or the median and interquartile range (IQR; non-parametric distribution).

The student t-test for paired samples was used to explore the differences in the TcpO_2_ values within the treatment with the sucrose octasulfate dressing because of the normal distribution of the variables. *p*-values < 0.05 were considered to be statistically significant, with confidence intervals of 95%.

## 3. Results

A total of 50 patients with non-infected neuroischaemic diabetic foot ulcers were included in the present study and followed for 20 weeks or until they were healed. The demographic characteristics, DM, and related foot complications at the baseline are shown in Table 1.

At the baseline, 20 (40%) patients had impaired microcirculation (tcpO_2_ < 30 mmHg), with mean tcpO_2_ values of 20.20 ± 5.38 mmHg at the moment of inclusion. Additionally, the forefoot was the most frequent location; 40 (80%) and 10 (20%) of the DFUs were in the rearfoot. The wound characteristics are shown in Table 2.

The TcpO_2_ values after TLC-NOSF dressing application showed an increase between day 0 and the end of the study in the whole population (33.04 ± 12.27 and 40.89 ± 13.06 mmHg, respectively) (*p* < 0.001). Additionally, when they were analyzed separately, both of the groups showed a local improvement in tissue oxygenation. The patients with impaired microcirculation showed an increase in the tcpO_2_ values from day 0 (20.20 ± 5.38 mmHg) to the end of the study (31.28 ± 13.74 mmHg) (*p* = 0.023) (Table 3). The patients with normal microcirculation also increased from 41.60 ± 6.80 mmHg at the point of inclusion to 46.73 ± 8.53 mmHg (*p* = 0.007).

From the 20 patients who had impaired microcirculation, at the end of the study, 13 (65%) patients achieved a normal microcirculation value (*p* < 0.001). Out of the whole study population, 13 (26%) patients did not achieve wound healing after 20 weeks of follow-up.

Furthermore, were observed a significant increase in the tcpO_2_ values in the forefoot DFU between day 0 (32.85 ± 12.76 mmHg) and until the wound closed (41.34 ± 12.02 mmHg) (*p* = 0.002) and in the rearfoot DFU between day 0 (33.80 ± 10.66 mmHg) and until the wound closed (39.25 ± 17.21 mmHg) (*p* = 0.071), with no difference between the ulcer locations (*p* = 0.694) (Table 4).

## 4. Discussion

The results of the present study confirm an improvement in the microcirculatory status derived from the sucrose octasulfate-impregnated dressings in the neuroischaemic DFU treatment. The transcutaneous oxygen pressure showed an increase in the local oxygenation during the wound healing. Additionally, this enhancement happened regardless of the vascular status at the baseline or the forefoot location. These findings indicate an added value to sucrose octasulfate dressings to support the first line of treatment in neuroischemic patients recommended by the IWGDF [22].

Several studies have explored potential therapies that could improve microcirculation due to the growing prevalence of PAD and compounds by the diffused nature of the vascular affection. Local therapies, such as hyperoxygenated fatty acids [8,9] and topical Vitamin E acetate [23], or physical procedures, such as whole body vibration [24], have recently documented their usefulness as a primary and secondary preventive tools in non-ulcerated diabetic patients.

On the same vein, some local and systemic treatments for DFU, such as low-intensity laser irradiation [10,25], injections of adipose-derived stromal vascular fraction cells [11], a systemic and local natural extract from the bark of the French maritime pine [12], the use of some heparins (e.g., dalteparin) [13], other systemic treatments with antiplatelets properties [26], or the use of local skin flaps [27] have been reported to produce a local increase in skin microcirculation.

Despite this, some of these are invasive processes or systemic treatments that need additional local wound care, thus increasing the direct costs. Finally, they used different methods to evaluate skin oxygenation. Consequently, it is difficult to compare these findings with the results of our study.

A previous pilot study [15] performed on a total of 11 patients with the same neuroischemic characteristics showed a local improvement in the tcpO_2_ values after using the sucrose octasulfate dressing between day 0 (29.45 ± 7.38 mmHg) and until the wound closed (46.54 ± 11.45 mmHg) (*p* < 0.016). Following the same trend derived from the current research, we observed an improvement from 33.04–12.27 mmHg at day 0 and 40.89–13.06 mmHg until the wound closed (*p* < 0.001). Thus, we could confirm the beneficial effect of this local procedure on the microcirculation, leading to an increase in tissue oxygenation.

Following this trend, another two therapies (systemic hyperbaric oxygen and autologous combined leucocyte, platelet, and fibrin) have reported promising results in the enhancement of the microcirculatory status. These therapies are recommended by the wound healing interventions guidelines (IWGDF), and they must be considered in non-healing ischaemic diabetic foot ulcers as adjunctive treatments [22].

Amir N. Wadee et al. [25] demonstrated a local improvement of the tcpO_2_ values by the treatment of chronic DFU with systemic hyperbaric oxygen. They found a significant increase between the baseline (20.26 ± 5.26 mmHg) and the three post-measures in the second (29.15 ± 5.78 mmHg), fourth (39.48 ± 8.43 mmHg), and sixth weeks (50.15 ± 11.13 mmHg) of the treatment (*p* = 0.000).

Moreover, Dubsky et al. [28] demonstrated an improvement of the tcpO_2_ values from 20.8 ± 9.6 to 41.9 ± 18.3 mmHg (*p* = 0.005) after 12 weeks of treatment with autologous cell therapy (ACT) in patients with diabetes and no-option chronic limb-threatening ischemia and foot ulcers, which are above those of the standard treatment (*p* = 0.034). However, this did not change significantly in the ACT group from 12 to 24 weeks.

Both of them reported similar results to ours in a similar population and with a similar measurement methodology of skin oxygenation and follow-up. Nevertheless, it is a fact that both of them are more expensive therapies with poor accessibility for this kind of population.

The growing interest in microcirculation studies in recent times is obvious and is reflected in the number of publications that try to demonstrate which therapies promote an effect on it.

The results of our study confirm the previously reported effect of sucrose octasulfate-impregnated dressings [15] on the microcirculation of neuroischemic patients. Microcirculation improvement could be related with mechanisms that sould be studied in further research.

Finally, our results should be interpreted with caution due to a major limitation: there is no control group for a comparison to be made. Therefore, further research should confirm the present results in a controlled and randomized clinical trial, in which the variables that could influence on peripheral oxygenation such as HbA1c or others must be evaluated. The main strength of the present study is that it is the first prospective study that demonstrates an improvement in the microcirculatory status in patients with impaired diabetic microcirculation at inclusion derived from the use of a dressing recommended by the IWGDF Guidelines in neuroischemic patients. These findings could help patients during and after the treatment to improve their microcirculatory status, even in the presence of satisfactory or delayed blood flows. The authors look forward to providing subsequent data from the 1 year follow-up prospective study after the patients have healed to analyze if a sustained improvement of the microcirculation could potentially have a positive effect on the recurrence rate of these lesions.

## 5. Conclusions

The local treatment with TLC-NOSF dressing improved microcirculation in patients with DFU regardless of their vascular status at the baseline or the forefoot location.

## Figures and Tables

**Table 1:** Demographics characteristics of patients at baseline.

Variables	Patients (*n* = 50)
Male, *n* (%)	45 (90%)
Female, *n* (%)	5 (10%)
Mean age ± SD (years)	62.60 ± 8.94
Type 1 diabetes, *n* (%)	4 (8%)
Type 2 diabetes, *n* (%)	46 (92%)
Glycated hemoglobin (%)	7.81 ± 1.47
Duration of diabetes ± SD (years)	20.04 ± 11.43
Risk factors	
Retinopathy, *n* (%)	18 (36%)
Nephropathy, *n* (%)	9 (18%)
Cardiopathy, *n* (%)	22 (44%)
Hypertension, *n* (%)	39 (78%)
Hypercholesterolemia, *n* (%)	28 (56%)
Tobacco use, *n* (%)	7 (14%)
Previous ulceration, *n* (%)	45 (90%)
Previous amputation, *n* (%)	40 (80%)
Vascular assessment	
History of revascularization, *n* (%)	16 (32%)
Bypass surgery, *n* (%)	3 (18.75%)
Endovascular surgery, *n* (%)	13 (81.25%)
Presence of dorsalis pedis pulse, *n* (%)	19 (38%)
Presence of posterior tibial pulse, *n* (%)	13 (26%)
Ankle brachial pressure index, mean ± SD	1.08 ± 0.36
Toe brachial pressure index, mean ± SD	0.69 ± 0.28
TcpO_2_ Day 0 (mmHg) ± SD	33.04 ± 12.27
Systemic antiplatelet treatments	35 (70%)

BMI, body mass index; TcpO_2_, transcutaneous oxygen pressure; SD, standard deviation.

**Table 2:** Wound characteristics of patients at baseline.

Wound Characteristics	Patients (*n* = 50)
Wound duration (weeks), median (IQR)	2.50 (2–8)
Wound area (cm^2^), median (IQR)	1.55 (1.20–2.35)
Pollina score, mean ± SD	4.60 ± 1.80
**University of Texas Diabetic Wound Grade Classification**	
IC: Ischemic, not infected, superficial wound, *n* (%)	44 (88%)
IIC: Ischemic not infected wound penetrating to tendon or capsule, *n* (%)	6 (12%)

IQR, interquartile range; SD, standard deviation.

**Table 3:** Differences in tcpO_2_ values in the feet of patients after sucrose octasulfate dressing application depending on microcirculation impairment (whole population and impaired microcirculation).

Visit	All Patients (*n* = 50)	*p* Value	Impairment Microcirculation Patients (*n* = 20)	*p* Value
Day 0	33.04 ± 12.27	-	20.20 ± 5.38	-
Week 4	33.87 ± 12.58	<0.001 *	26.53 ± 10.21	0.002 *
Week 8	30.60 ± 11.83	0.402	24.67 ± 10.02	0.390
Week 12	44.30 ± 11.79	0.046 *	41.50 ± 7.77	<0.001 *
Week 16	44.85 ± 5.89	<0.001 *	44.50 ± 12.02	<0.001 *
Week 20	49.50 ± 2.12	<0.001 *	51.00	-
Wound closure	40.89 ± 13.06	<0.001 *	31.28 ± 13.74	0.023 *

* Differences were assumed significant at *p* < 0.05 for a confident interval of 95%.

**Table 4:** Differences in tcpO_2_ values in the feet of patients after sucrose octasulfate dressing application depending on DFU location (forefoot and rearfoot).

Visit	Forefoot Location (*n* = 40)	*p* Value	Rearfoot Location (*n* = 10)	*p* Value
Day 0	32.85 ± 12.76	-	33.80 ± 10.66	-
Week 4	34.69 ± 13.57	<0.001 *	30.85 ± 7.9	0.914
Week 8	29.29 ± 10.82	0.277	34.33 ± 14.8	0.007 *
Week 12	44.16 ± 10.34	0.287	44.50 ± 15.45	0.523
Week 16	47.6 ± 4.15	0.854	38.00 ± 2.82	<0.001 *
Week 20	48.00	-	51.00	-
Wound closure	41.34 ± 12.02	0.002 *	39.25 ± 17.21	0.071

* Differences were assumed significant at *p* < 0.05 for a confident interval of 95%.

## Data Availability

The data are available previous request to corresponding author.
